# A Melanoma Lymph Node Metastasis with a Donor-Patient Hybrid Genome following Bone Marrow Transplantation: A Second Case of Leucocyte-Tumor Cell Hybridization in Cancer Metastasis

**DOI:** 10.1371/journal.pone.0168581

**Published:** 2017-02-01

**Authors:** Greggory S. LaBerge, Eric Duvall, Zachary Grasmick, Kay Haedicke, John Pawelek

**Affiliations:** 1 Human Medical Genetics and Genomics Program, University of Colorado School of Medicine, Aurora, CO, United States of America; 2 Denver Police Crime Lab-Forensics and Evidence Division, Denver, Colorado, United States of America; 3 Department of Pathology, University of Colorado AMC, Denver, Colorado, United States of America; 4 Department of Internal Medicine Section of Medical Oncology and the Yale Cancer Center, Yale School of Medicine, New Haven, Connecticut, United States of America; 5 Department of Dermatology and The Yale Cancer Center, Yale School of Medicine, New Haven, Connecticut, United States of America; Universitat Witten/Herdecke, GERMANY

## Abstract

**Background:**

Metastatic disease is the principal cause of mortality in cancer, yet the underlying mechanisms are not fully understood. Macrophage-cancer cell fusion as a cause of metastasis was proposed more than a century ago by German pathologist Prof. Otto Aichel. Since then this theory has been confirmed in numerous animal studies and recently in a patient with metastatic melanoma.

**Methods:**

Here we analyzed tumor DNA from a 51-year-old man who, 8 years following an allogeneic BMT from his brother for treatment of chronic myelogenous leukemia (CML), developed a nodular malignant melanoma on the upper back with spread to an axillary sentinal lymph node. We used laser microdissection to isolate FFPE tumor cells free of leucocytes. They were genotyped using forensic short tandem repeat (STR) length-polymorphisms to distinguish donor and patient genomes. Tumor and pre-transplant blood lymphocyte DNAs were analyzed for donor and patient alleles at 15 autosomal STR loci and the sex chromosomes.

**Results:**

DNA analysis of the primary melanoma and the nodal metastasis exhibit alleles at each STR locus that are consistent with both the patient and donor. The doses vary between these samples indicative of the relative amounts of genomic DNA derived from the patient and donor.

**Conclusion:**

The evidence supports fusion and hybridization between donor and patient cells as the initiator of metastasis in this patient. That this phenomenon has now been seen in a second case suggests that fusion is likely to play a significant role for melanoma and other solid tumor metastasis, perhaps leading to new avenues of treatment for this most problematic disease.

## Introduction

Leucocyte-tumor cell fusion and hybridization as a mechanism of solid tumor metastasis was first proposed by Aichel more than a century ago [1]. Decades later the idea resurfaced with similar proposals from several sources [2–13]. While fusion and hybridization as a mechanism of metastasis has been well demonstrated in animal models, less is known about the phenomenon in humans [11]. In previous studies with two patients who each received allogeneic bone marrow transplants then later developed renal cell carcinomas, donor alleles were found in patient tumor cells, suggesting fusion had occurred. But in these studies there was no provision to identify patient alleles in the tumor and fusion could not be confirmed [14,15]. More recently we used STR length-polymorphisms to genotype a melanoma metastatic to the brain in a patient who 6 years previously had received an allogeneic BMT [2]. In 9 histologic sections from one end of the tumor to the other, all alleles in the donor and patient pre-BMT lymphocytes were found in tumor cells. The allelic ratios were similar throughout, indicating that the tumor was likely generated from a clone through a single fusion-hybridization event. This constituted the first evidence for leucocyte-cancer cell hybrid formation as a mechanism of metastasis in humans.

Here we analyzed tumor biopsies from a second patient who had also received an allogeneic BMT and later developed malignant melanoma with metastases. Using STR length-polymorphisms and forensic genetic techniques we analyzed genomic DNA from a primary melanoma on the upper left back and compared it to an axillary lymph node metastasis. Both the primary melanoma and secondary metastasis contained a mixture of donor and patient DNA, again implicating cell hybridization as the cause of metastasis. That hybrids have now been found in a second patient suggests that the phenomenon is likely to be widespread and could point the way for the design of new therapies for metastatic disease, the main cause for mortality in cancer.

## Methods

### Ethics statement

All samples used in this study were preexisting and de-identified before being received by the Yale research team. Exemption was granted under Yale IRB protocol #070900309 (JP) from the Yale University Human Research Protection Program, Institutional Review Board.

### Source of Tissues

The patient was a 51-year-old man who received an allogeneic BMT from his brother for treatment of chronic myelogenous leukemia (CML). Eight years later he developed metastatic melanoma at multiple sites. We received samples of the primary tumor and a left axillary lymph node metastasis that were fixed in formalin and embedded in paraffin (FFPE) by standard histological procedures. Pre-transplant donor and patient lymphocytes were stored at -90°C in the Yale-New Haven Hospital Stem Cell Bank.

### Immunohistochemistry

Slides were stained with Anti-S100 (DAKO, IVD IVD IR504, Rabbit Polyclonal) and photographed with a Zeiss Axioskop 40 light microscope equipped with a Spot Flex digital camera.

### Laser Microdissection

Laser Microdissection was as described previously (2). Handling and processing of FFPE tissue samples was carried out using ultraclean, DNA-free equipment. Five μicron-thick histological sections were cut and immunostained for Leucocyte Common Antigen (LCA/CD45) (clone 2B11 + PD7/26, Dako, catalog N1514) using an autostainer (DAKO, Carpinteria, CA). Tumor cells were microdissected free of LCA/CD45-positive cells using an Arcturus XT laser dissection microscope system. Each sample consisted of dissected tumor cells pooled from one or more areas of the same histological section into a single tube. About 500–1000 cells were isolated from the primary tumor and 1500–2000 from the lymph node metastasis.

### DNA extraction

DNA extraction and STR analyses were by the Denver Police Department Crime Laboratory DNA Unit using validated forensic standard operating procedures [16,17]. Samples were collected into GeneAmp thin-walled reaction tubes (0.5 ml; Applied Biosystems, Carlsbad, CA, USA). Lymphocyte DNA was extracted manually using the FFPE Tissue kit (Qiagen in USA). Total human and male DNA were assessed with the Quantifiler Trio DNA Quantification Kit (Applied Biosystems, Carlsbad, CA, USA). DNA was extracted using the RecoverAll Total Nucleic Acid Isolation Kit for FFPE Tissues (Applied Biosystems, Carlsbad, CA, USA). The number of tumor cells microdissected per sample was automatically recorded by the Arcturus XT system.

### PCR amplification

PCR was performed with the AmpFlSTR IdentifilerPlus PCR Amplification Kit (Applied Biosystems, Carlsbad, CA, USA). Generally, 1.4 ng of total DNA was targeted in each PCR amplification. In samples with less DNA (< 0.1 ng/μl), samples were concentrated 4 fold using a Microcon centrifugal filter (Ultracel Ym-100, Millipore, Billerica, MA, USA).

### Forensic genetic analyses of Short Tandem Repeat-STR loci

STR loci have been selected to be neutral with respect to other genetic linkage or associations with either Mendelian or non-Mendelian disorders. The loci are polymorphic and exhibit acceptable levels of heterozygosity, typically 70% or higher. They are assayed together as a PCR multiplex and are robust for degraded DNA [16,17]. Genotyping of PCR products and interpretation of STR alleles was performed using capillary electrophoresis on an ABI Prism 3130 Genetic Analyzer with GeneMapper ID Software version 3.2 (Applied Biosystems, Carlsbad, CA, USA). X,Y chromosomes were detected using the Amelogenin assay concurrent with the autosomal STR analyses [16,17]. Qualitative and quantitative signal-to-noise thresholds were determined with the ABI Identifiler Kit in previous validation studies by the laboratory. All peaks greater than the validated dye channel specific analytical thresholds 23–40 RFU (relative fluorescence units) were scored as true alleles based on a) height and b) peak morphology (S1 File http://www.cstl.nist.gov/strbase/multiplx.htm [16,17].

### Allelic stutter in mixed DNA samples

In DNA samples with more than one individual’s genomic DNA, peaks from one individual sometimes overlap stutter positions of alleles from another individual. Based on laboratory validation studies and those of others, interpretation guidelines have been established that allow whether a given peak is interpreted as an allele or as potential stutter PCR artifact for any given allele at any locus [18,19]. All the alleles deemed significant in this study conformed to these guidelines and were not deemed to be stutter artifacts.

## Results

**Fig 1:**
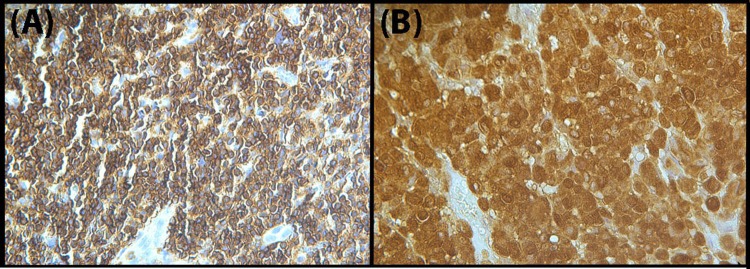
Photomicrographs of the primary melanoma (A) and the lymph node metastasis (B) studied herein and stained with anti-S100.

Fig 1 shows an immunohistochemical analysis of the primary (A) and metastatic (B) tumor stained with anti-S100 protein. Tumor cells stained brown and lymphocytes stained blue. The primary tumor consisted of a mixture of cancer cells and tumor infiltrating lymphocytes while in the metastatic tumor the preponderance of cells were cancer calls and lymphocytes were seen only occasionally. This is in line with numerous reports that metastatic tumors express mechanisms for evading the immune system [20]. Further, the metastatic cells (B) expressed far more S100 protein than did the primary tumor cells (A). In tis regard, S100 is associated with decreased survival times in patients with metastatic melanoma [21].

Tumor cells were dissected free from leucocytes by laser microdissection and DNA was extracted. STR analyses were performed of DNA at 15 autosomal loci and the sex chromosomes to look for BMT donor and patient DNA in the patient’s primary tumor and an axial lymph node metastasis. In both the primary tumor and lymph node metastasis, alleles were scored that are consistent with both the patient and donor across all the STR loci (Table 1). The proportionate amounts of genomic DNA differ between the primary tumor and lymph node metastasis. The PCR based STR assay is semi-quantitative in nature and our results indicate that in both tumor sources that alleles consistent with the patient are in abundance to those alleles consistent with the allogenic bone marrow donor. In the primary tumor, the patient alleles are approximately five times more abundant than donor alleles based on relative fluorescence units on the ABI Genetic Analyzer and within interpretation thresholds. Within the lymph node metastasis, the primary source of DNA is the patient but the donor is also represented across the STR loci genotyped. In these samples, the patient genomic DNA is approximately ten to fifteen-fold in abundance to the donor DNA where peaks corresponding to alleles consistent with the bone marrow donor are much lower and hence less abundant. Thus both the primary tumor and the metastasis contained genomic DNA consistent with the genomes of both the patient and donor.

**Table 1:** STR genotyping of DNA from donor (D), patient (P), primary tumor and lymph node metastasis. STR units: number of tandem repeats of the locus-specific tetranucleotide sequence.

STR Locus	Primary Tumor	Lymph Node	Patient Sample	Donor Sample
D8S1179	13,15	13,15	13,15	13
D21S11	28,29,30,30.2	28,29,30,30.2	28,29	30,30.2
D7S820	11,12	11,12,14	12,14	11
CSF1PO	9,11	10,11,12	11,12	9,10
D3S1358	15,16,18	16,18	16,18	15,16
TH01	6,7,9	6,7,9	6	7,9
DS13S317	8,9,12	8,12	12	8,9
D16S539	13	11,13	11,13	13
D2S1338	16,17,18	17,19	17,19	16,18
D19S433	13,15,16	13,15,16	15,16	13,16
vWA	17,18,19	17,18	17,18	18,19
TPOX	8,9,11	8,9	8,9	8,11
D18S51	12	12,20	12,20	15,18
Amelogenin	X,Y	X,Y	X,Y	X,Y
D5S818	9,11,12	9,11,12	11	9,12
FGA	21,22,24	21,24	21,24	22,25

## Discussion

Here we found a mixture of donor and patient DNA in a metastasis from a second patient who had earlier received an allogeneic BMT, strongly indicating that the metastasis was a hybrid. That this is the second such case suggests that hybridization is likely to play a significant role in tumor progression [2]. The fusion and hybridization process is not understood. As suggested previously, perhaps a macrophage or other phagocyte does not properly digest the cancer cell and the two cells fuse, pooling genetic material into a single nucleus and generating a white blood cell-cancer cell hybrid. These cells would become metastatic when they expressed both the uncontrolled cell division of the cancer cell and the chemotactic motility of phagocyte [22]. It is not known whether the primary tumor needs to be actively dividing or perhaps indolent for fusion to occur. It is also possible that fusion of melanoma cells occurred with stromal cells such as fibroblasts as has recently demonstrated in vitro [23,24]. Indeed, fibroblasts can respond to chemotactic cues and, like macrophages, could in theory confer chemotactic motility on fibroblast-cancer cell hybrids [23–25].

We used the same PCR/STR system in this study that we used in our previous study [2], namely Identifiler kit from Applied Biosystems that has markers on chromosomes: 8, 21, 7, 3, 13, 16, 2, 19 18, 5, 11, 12, 4, X, Y. Certainly adding more markers on more chromosomes would yield additional information regarding genome instability and can be explored in further research, possibly using extensive SNP panels to cover most of the genome. This work would require much more DNA than we have been able to isolate from FFPE tissue even though the forensic kits used are optimized to provide results with low amounts of DNA that is often degraded in picrogram amounts. Adding markers with other forensic kits could be informative but would most likely provide the same conclusions as we currently have, as the discriminating power of the Identifiler STR system is very high.

Leucocyte-tumor cell fusion provides a unifying explanation for metastasis. While primary tumors arise in a wide variety of tissues and are not a single disease but many different diseases originating from many different mutations, metastatic cancer may be only one disease arising from a common, non-mutational event—fusion of primary tumor cells with myeloid cells. Indeed, metastatic cancers and myeloid cells share many traits in common [26–27]. This could make metastatic cells more amenable to the design of new therapies independent of the origin of the primary tumor [22]. For example, BMDC-cancer cell fusion as a source of metastatic cells would imply that prevention of fusion or of rate-limiting post-fusion events such as pathways governing the integration of parental fusion partner genes into hybrid genomes, or with activation of regulatory genes that control cell migration. Cells in the act of fusing, or post-fusion cells might have unique antigenic profiles, making them targetable through immunotherapy.

Finally, the findings confirm Aichel’s remarkable proposal from 1911 that the source of aneuploidy in metastatic tumors is caused by “fusion of tumor-invading macrophages with tumor cells that creates qualitative differences in chromosomes from the two cell types, leading to the metastatic phenotype” (translation from German) [1]. Our results suggest that research focused on such pathways could produce new techniques for targeting the metastasis process itself, perhaps preventing this deadly phenomenon [28,29].

## Supporting Information

S1 File http://www.cstl.nist.gov/strbase/multiplx.htm. (DOCX)
