# Supplementary material for: A Melanoma Lymph Node Metastasis with a Donor-Patient Hybrid Genome following Bone Marrow Transplantation: A Second Case of Leucocyte-Tumor Cell Hybridization in Cancer Metastasis
Source: PLoS One. 2017 Feb 1;12(2):e0168581. doi: 10.1371/journal.pone.0168581 (PMC5287451; doi:10.1371/journal.pone.0168581)
Supplement: S1 File — (DOCX) [file pone.0168581.s001.docx]

**Supporting Information:** File S1.

[**http://www.cstl.nist.gov/strbase/multiplx.htm**](https://urldefense.proofpoint.com/v2/url?u=http-3A__www.cstl.nist.gov_strbase_multiplx.htm&d=CwMGaQ&c=-dg2m7zWuuDZ0MUcV7Sdqw&r=UaN7wooB53uozqN07iUw6l5mgnJvFBN3lfO06u4ETZ8&m=O7UhTZBy8Kg6cXDCBmQ-jyNfl0KWO_iLbtV0oPCjgAo&s=Yml2BF6HTsUlvvbY0JWrOORbqSBXW5QOBNXpDgRYLuQ&e=).
